# Personality traits and resilience mediated by depressive symptoms in a sample of psychotherapy patients: A cross-sectional study

**DOI:** 10.1371/journal.pone.0327846

**Published:** 2025-08-06

**Authors:** Leonardo Gonçalves, Vitória Maria Tabosa Evaldt, Neusa Sica da Rocha

**Affiliations:** 1 Post Graduate Program in Psychiatry at Federal University of Rio Grande do Sul (UFRGS), Porto Alegre, Rio Grande do Sul, Brazil; 2 Clinical Research Center and Psychiatry Service, I-Qol, Hospital de Clínicas de Porto Alegre (HCPA), Porto Alegre, Rio Grande do Sul, Brazil; School of Nursing Sao Joao de Deus, Evora University, PORTUGAL

## Abstract

**Introduction:**

Resilience is a concept that has been widely studied in different contexts and is related to adaptation to different stress levels. Although research in psychotherapy has produced vast knowledge about the effectiveness and mechanisms that mediate response, there is still no clarity about the role of resilience in this process, especially research in psychotherapy with patients with severe mental illness. Due to the implications of severe mental illness in different areas of life, in these patients, the capacity for resilience may be impaired, and the mechanisms involved in mediating this process are still not well explained.

**Objective:**

Verify the associations between personality traits and resilience and their potential confounders (sociodemographic factors, pharmacological treatment, type of psychotherapy, social support, depressive and anxiety symptoms). Assess depressive symptoms as a possible mediator of the relationship between personality traits and resilience.

**Methods:**

This is a naturalistic cross-sectional study, with 83 participants undergoing psychotherapy from different theoretical lines (psychodynamic, cognitive-behavioural, and interpersonal). Sociodemographic characteristics were evaluated, as well as resilience (25-item Connor Davidson Resilience Scale), depressive symptoms (Beck Depression Inventory), anxious symptoms (Beck Anxiety Inventory), and Personality traits (Personality Inventory for DSM-5).

**Results:**

Resilience was negatively associated with depressive symptoms (B = −12.1, p < 0.001). The mediation effect was significant for depressive symptoms (b = −7.21, BCa 95% CI [−12.6, −3.07]). This variable mediated approximately 46% of the relationship between negative affectivity and resilience.

**Conclusion:**

Resilience was positively associated with antagonism traits and negatively with negative affectivity and depressive symptoms. Depressive symptoms mediated 46% of the relationship between negative affectivity and resilience. The main findings of this study contribute to a greater understanding of the mechanisms involved in resilience, thus influencing the mental health outcomes of patients with severe mental illness.

## Introduction

In recent decades, there has been a growing interest in the concept of resilience related to mental health, and extensive literature has been published [1]. The first studies about resilience dated from the 1970s on the development of at-risk children who became healthy adults despite adversity, seeking to understand the risk and protection factors. In the second wave, resilience was understood as a disruption-reintegration process, and the qualities involved in this process were sought [2]. The third and contemporary wave defines resilience as a multidisciplinary construct of a dynamic process of successful adaptation to different stress levels [3].

There is no consensus regarding the definition of the resilience concept, with each research area using its own according to the focus of the study, be it at-risk population such as the military, university students, chronic patients, or in the recovery of trauma victims [4]. Several scales have also been developed in the last few decades to measure resilience and understand its mechanisms in the above mentioned situations. The lack of conceptual standardization has generated criticism regarding the quality and reproducibility of studies, and recently, some authors have proposed a more rigorous methodology for new publications in this field [5–7].

Despite the extensive literature on the effectiveness and mediating mechanisms of response in the various existing psychotherapies, there is no clarity as to the role of resilience in this process. In recent years, some intervention studies on resilience, with mindfulness and cognitive-behavioral therapy, have shown that they are the most significantly effective [8]. However, there is still a scarcity of longitudinal studies prospectively evaluating the impact of resilience on the mental health of both the clinical and community populations at risk of developing some psychopathology [9,10]. These studies, despite methodological limitations, show that resilience could act as a “buffer” for anxious and depressive symptoms after stressful events in healthy populations. As for clinical populations, we have less data, the majority of which is in the treatment of post-traumatic stress disorder (PTSD) [11].

Resilience has been less studied in the population of patients with severe mental disorders, which refers to patients with chronic mental disorders with high functional impairment [12–14]. The association of resilience with clinical measures in hospitalized patients diagnosed with severe mental disorders: major depression, bipolar disorder, and schizophrenia, when comparing resilience levels among these different disorders (schizophrenia, bipolar disorder, and major depression), showed that patients with major depression had lower levels of resilience compared to those diagnosed with the two other disorders [15]. These patients have high rates of unemployment, social isolation, suicide attempts, and psychiatric hospitalizations. They are also exposed to different types of acute and chronic stressors at various levels, resulting in complex psychopathology and greater use of health services. These patients are generally seen in psychiatric outpatient clinics and require combined drug and psychotherapeutic treatments. In this population, where the capacity for resilience seems to be impaired, the processes involved and their mediating mechanisms are not well elucidated [13].

According to Windle, the protective factors related to resilience can be divided into three different levels of functioning: 1) individual, 2) social, and 3) community [16]. In this paper, we focused on individual factors, such as sociodemographic and mainly psychological or intrapsychic (personality traits) factors, and social or external factors, such as social support and social stressors, by involving patients in psychotherapeutic treatment. Other factors related to resilience were included, such as the type of psychotherapy and pharmacological treatment.

The main objective of this study is to verify the associations between personality traits and resilience and their potential confounders (sociodemographic factors, pharmacological treatment, type of psychotherapy, social support, depressive and anxiety symptoms). The secondary objective is to assess depressive symptoms as a possible mediator of the relationship between personality traits and resilience.

Primary hypothesis: there is a positive association between personality traits and resilience. Anxious and depressive symptoms may mediate this association. Higher levels of depressive and anxiety symptoms and psychosocial stressors are associated with lower levels of resilience.

Secondary hypothesis: Older age, higher levels of education, and male gender are associated with higher levels of resilience. More significant social support, pharmacological treatment, and cognitive-behavioral therapy are also associated with higher levels of resilience.

## Methods

This study is part of a larger project called Longitudinal Investigation of Psychotherapy Outcomes (LIPO), and its research protocol has been described in greater detail elsewhere [17].

### Design

The present study is a naturalistic cross-sectional study, and its population corresponds to the baseline sample of the larger project mentioned above. The primary study is a longitudinal observational study with a 12-month follow-up (baseline, 06 months, and 12 months) of outpatient follow-up in three types of psychotherapy: psychodynamic psychotherapy (PDT), cognitive-behavioral psychotherapy (CBT), and interpersonal psychotherapy (IPT). The data were collected between March 2015 and December 2017.

### Sample and procedures

The sample of this study is composed of 83 patients who underwent one of three evidence-based modalities of individual psychotherapy [psychodynamic psychotherapy (PDT), interpersonal psychotherapy (IPT), and cognitive–behavioral psychotherapy (CBT)], with age above 18 years old who started the psychotherapy within one month and had a Beck Depression Inventory (BDI) score greater than 15. Patients with psychotic disorders, in current use of psychoactive substances (alcohol, cocaine, marijuana. etc.), cognitive deficits, and dementias were excluded. This sample corresponds to outpatients referred from a screening outpatient clinic that attends the other clinical outpatients of a general hospital (Hospital de Clínicas de Porto Alegre).

Indication of the psychotherapy modality considered previous favorable experience with some psychotherapy, psychological-mindedness, presence of personality disorder or traits, number of diagnoses of psychiatric disorders, and relief of symptoms. When diagnosed with a personality disorder associated with one or more major psychiatric disorders and psychological-mindedness, PDT was indicated. When there are up to two primary psychiatric diagnoses without personality disorder, it will be directed to CBT as well as in the exclusive quest for symptom relief. When the focus of the problem was exclusively up to two of the following: 1) dispute, 2) roles dispute, 3) role transition, or 4) interpersonal deficits and no personality disorder, IPT was indicated. Psychiatry residents provided psychotherapies under weekly supervision.

### Instruments

#### Resilience.

Connor-Davidson Resilience Scale (CD-RISC) developed by Connor & Davidson: measures levels of positive psychosocial adaptation in the face of significant life events. Comprises 25 items with a Likert response ranging from 0, “not at all true,” to 4, “almost always true” [18].

#### Personality.

The Personality Inventory for DSM-5 (Diagnostic and Statistical Manual of Mental Disorders) (PID-5) – Adult. A set of 25 core elements of personality description that combine into five broad domains of maladaptive personality variation: negative affect, detachment, antagonism, disinhibition, and psychoticism [19].

#### Psychosocial stressors.

Life Events Questionnaire (LEQ): assesses the occurrence of 14 stressful life events in the last 12 months, their impact on the subject’s life, and the association with the onset of current psychiatric problems [20].

#### Symptoms of anxiety and depression.

Beck Depression Inventory (BDI) and Beck Anxiety Inventory (BAI): They are self-report scales of the magnitude of depression (BDI) and anxiety (BAI) developed by Beck, aiming to evaluate levels of anxiety, suicidal ideation, and depression [21].

#### Social support.

Social Support Questionnaire of the MOS Study: This questionnaire was developed to assess the social support received by patients with chronic diseases and adapted to Portuguese [22]. It consists of 19 self-report items and a Likert format with a score of 1–5, in which the higher the score, the greater the social support [23,24].

### Statistical analysis

Continuous sociodemographic variables were presented as means and standard deviations (SD), and categorical variables were presented as percentages. Pearson’s bivariate correlation was used for the correlation test, and linear regression with the backward method was used for multivariate analyses. Factors that had a previous association with resilience, according to the literature, were included in the statistical model: sociodemographic [25], social support [26,27], pharmacological treatment [11,28], type of psychotherapy [8,29], psychosocial stressors [10], depressive and anxiety symptoms [30–32] and personality traits [33–36]. The level of significance was set at p < 0.05.

The mediation analysis was performed with the statistically significant factors obtained in the logistic regression using the bootstrapping method described by Preacher and Hayes [37], To understand the impact of personality traits on resilience (negative affectivity), a mediation analysis was performed using depressive symptoms as a mediator, as they are a state variable. To understand the finding of the antagonism trait positively impacting resilience, a multivariate regression analysis was performed using only the facets of this trait to determine which ones influenced resilience.

All analyses were performed using SPSS version 21.

### Ethical aspects

As previously mentioned, this study is part of the LIPO project, approved by the Research Ethics Committee of Hospital de Clínicas de Porto Alegre (GPPC—HCPA number 150097). All study participants signed an informed consent form outlining the study’s objectives. No research team members participated in the clinical and psychotherapeutic treatment of patients.

## Results

Table 1 shows the characteristics of the sample. Furthermore, in relation to psychotherapeutic treatments, the main indication for psychotherapy was for PDT (57.8%), with more than double that of the second, which was CBT (24.1%), and lastly, IPT (18.1%). It is noteworthy the high prevalence of medication use (90%) as well as suicide attempts (39.4%) and recent psychiatric hospitalization (28.2%), denoting a severe clinical population. Furthermore, the total CD-RISC mean score was 49.85 (SD:17.6), and the mean depression score, evaluated with BDI, was 30.43 (SD: 10.78).

**Table 1:** Characteristics of the sample.

	Total (n = 83)
**Mean Age (±DP)**	45.13 (12.38)
**Sex n (%)**	
Female	63 (77.8)
**Ethnicity, n (%)**	
White	72 (86.7)
Non-White	10 (12)
**Years of Study, mean (±DP)**	12,27 (4.06)
**Marital status n (%)**	
Single	20 (27.4)
Married	40 (54.8)
Divorced	9 (12.3)
Widowed	4 (5.5)

There was a positive correlation between resilience and social support (r = 0.321, p = 0.001), but no correlation was found between resilience and the stressors scale (r = 0.02, p = 0.66). We found a high negative correlation with depressive (r = −0.57, p = 0.01), anxious symptoms (r = −0.46, p = 0.01), and negative affectivity (r = −0.48, p = 0.01).

In the multivariate analysis (Table 2), factors with p ≤ 0.05 of the bivariate correlation analysis were inserted in the model (gender, suicide attempt, pharmacological treatment, depressive symptoms, anxiety symptoms, social support, negative affectivity, detachment and disinhibition). A statistically significant positive association was found with the trait of antagonism (B = 7.67, p = 0.006). Resilience was negatively associated with depressive symptoms (B = −0.5, p = 0.004) and negative affectivity (B = −12.1, p < 0.001). There was a positive association trend with pharmacological treatment, but without reaching statistical significance (B = 9.7, p = 0.07). The proportion of adjusted variability of resilience explained by the final model was 52% (R^2^_adj_ 0.52, p = 0.001).

**Table 2:** Multivariate adjustment of significant factors from the bivariate analysis associated with resilience.

	n	B (95% CI)	p-value
Pharmacological treatment	83	9.7 (−0.88–20.28)	0.072
BDI	83	−0.5 (−0.83 – −0.17)	0.004*
PID-5: Negative Affect	83	−12.1 (−17.9– −6.24)	<0.001*
PID-5: Antagonism	83	7.67 (2.29–13.04)	0.006*

*; R^2^ 0.55, adjusted R^2^ 0.52, p < 0.05. Factors included in the model: gender, suicide attempt, pharmacological treatment, depressive symptoms, anxiety symptoms, social support, negative affectivity, detachment and disinhibition.

**Fig 1:**
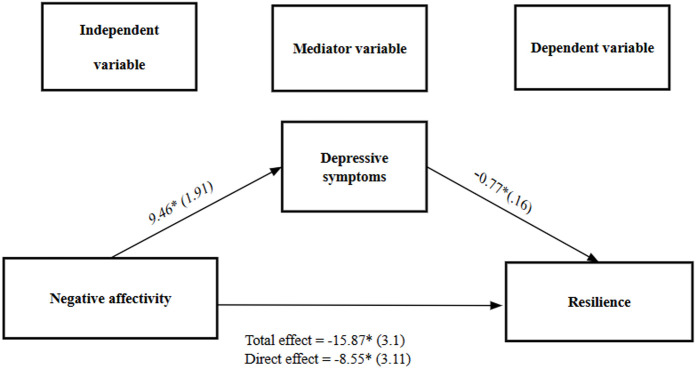
Negative affectivity as a predictor of resilience mediated by depressive symptoms. **(A)** The Bias-Corrected and Accelerated (Bca) confidence interval was estimated using Boostraping technique (5,000 resamples) **(B)** All coefficients represent unstandardized beta coefficients (standard errors in parentheses) **(C)** *p < 0.001.

Fig 1 shows that depressive symptoms [BDI] (b = −7.32, BCa Cl 95% [−12.6, −3.07]) were significant mediators, explaining approximately 46% of the relationship between negative affectivity [PID-5] and resilience [CD-RISC]. Only the grandiosity facet was significant (B = 7.29, p = 0.038). The other facets (dishonesty, seeking attention, manipulation, and insensitivity) did not show a significant association.

## Discussion

### Primary findings

This is the first study to our knowledge that investigates the factors associated with resilience in outpatients with severe mental disorders using different methods of psychotherapy (PDT, CBT, and IPT). The main objective of this study was to examine the association between personality factors and resilience. A negative association was found between negative affectivity and resilience, and a positive association between antagonism and resilience.

The PID-5 negative affectivity domain is analogous to the Big Five neuroticism construct in non-clinical populations and relates to the experience of negative emotions, including a study showing activation patterns in different regions of the neocortex than in a healthy population [38,39]. Both are associated as a risk factor for the development of depressive and anxious psychopathology in several studies [34–36,40,41]. It is not surprising that we find in a population of chronically ill patients the impact that this facet of personality can have on their ability to deal with various forms of stress, especially disorders such as depression, resulting in a reduced perception of resilience and, therefore, mitigation of these conditions and restoring their basal functioning, which would be a successful process for someone resilient [3,15].

This is evident with the mean level of resilience of this population of 49.85, comparable with other studies with clinical samples [42,43] but much lower than that of community populations with an average that varied between some studies compiled by the scale’s developers between 65.4 and 80.4 in different countries. In the work by Solano (2012) with a Brazilian sample of 108 outpatients, a mean value of 57.7 was found for patients with anxious symptoms and 52.1 for patients with borderline personality disorder [43]. Min et al. (2013) found values of 47.4 (20.2) in 230 outpatients with anxiety and depression [44], and Um et al. (2014) reported a mean of 46.0 (20.8) for 254 outpatients with depression [45]. A more recent study showed that patients with major depression have lower levels of resilience than even patients with schizophrenia and bipolar disorder [15].

In addition, we found an unprecedented association in the literature of the PID-5 antagonism domain on resilience. To understand this result, we analysed the facets that comprise the antagonism trait and found only grandiosity as a predictor of resilience. This finding might be, unfortunately, a result of a distorted view of these patients regarding their resilience. In the study by Nunes (2022), with inpatients but with a similar profile of high severity, it was found that resilience was comparatively lower in depressed patients compared to patients with schizophrenia and bipolar mood disorder [15].

### Secondary findings

To understand the relationship between trait and state, a mediation analysis was performed to clarify how much of the perception of patients in the sample of patients with negative affectivity influenced their resilience through their depressive symptomatology. It was observed that approximately 46% of this relationship was mediated by the depressive state (depressive symptoms). This result shows a complex interrelationship between personality (trait), symptomatology (state), and resilience, impacting overall mental health. Some authors consider that resilience could act as a buffer for the adverse effects of personality on symptoms of depression and anxiety [11].

For other factors that have already been extensively studied, such as social support and the level of psychosocial stressors, we found a positive correlation between resilience and social support. We had no statistically significant association with live stressors. The type of psychotherapy assigned was also not associated with the level of resilience.

This work sought to establish relevant study factors to be researched in assessing contributors to resilience in clinical populations with chronic and severe disorders. The field of resilience has developed mainly in the investigation of factors that protect mental disorders in populations at risk, such as health professionals, police, students and athletes [4]; however, it lacks a better understanding of the factors that may promote the restoration of at least partial resilience capacity in populations that already have functional chronic impairment and that are possibly unable to return to functioning at a healthy level, resulting in a lossy or dysfunctional reintegration [3]. By understanding the factors that promote resilience more effectively and those that reduce it, we can establish more specific interventions in psychotherapy, advancing not only in relieving symptoms.

### Limitations

This study has some limitations. This is a cross-sectional analysis of a baseline sample from a more extensive longitudinal study [17] with a 12-month follow-up, so it is impossible to infer causality in the associations found between negative affectivity, antagonism, depressive symptoms, and resilience. The generalization of its results to other populations is also limited. The sample studied, being evaluated in tertiary-level psychiatric outpatient clinics, is characterized by presenting what the World Health Organization considers as a severe mental disorder (SMD), that is, patients with high levels of psychopathology, a high number of suicide attempts, and hospitalizations. The field of resilience has been less focused on the investigation of populations with chronic mental disorders, where the capacity for resilience is reduced, so we believe that research on mechanisms that enable the promotion of resilience in patients with high demand for mental health services is valid.

There still needs to be more consensus in the literature regarding the definition of resilience or how best to evaluate it [4,16,46]. Several instruments aim to measure resilience with good psychometric properties without a gold standard [47]. CD-RISC was used because it was developed in patients with PTSD, which is like the severity of our sample, and because it is more sensitive to change due to treatment since this study is the baseline sample of a longitudinal study [17]. Other authors argue that questionnaires measure factors linked to resilience but not their underlying mechanisms [7,48]. They propose that resilience be measured quantitatively in longitudinal studies through the ratio of the load of mental health problems to the load of stressors [7]. Patients who presented no or minimal worsening of symptoms after one or more stressors would be resilient [48]. Studies of this nature require several measures over time, which makes them highly challenging. Measuring stressors in chronically ill patients becomes difficult, as there is no clear separation between the effect of the stressor and the onset of symptoms. In addition, self-administered questionnaires may present biases related to social desirability and memory bias, so there is still a lack of more objective measures of resilience.

### Future directions

The analysis of this sample’s follow-up may clarify the direction of the causality of the associations between negative affectivity, antagonism, depressive symptoms, and resilience shown in this study.

## Conclusion

The present work showed significant associations between personality traits of negative affectivity and antagonism and depressive symptoms and social support with resilience. Depressive symptoms mediated nearly half the relationship between negative affectivity and resilience. These findings contribute to a greater understanding of both the mechanisms that promote resilience and those that can reduce it, thus influencing the mental health outcomes of outpatients in different psychotherapies.

## Supporting information

S1 Protocol Description of the study protocol, April-2019. (PDF)

S2 Database Partial database including the main data of the mentioned instruments. (XLSX)
